# Risk Factors for Adverse Events in the Fontan Population: Does Ventricular Morphology Play a Role?∗

**DOI:** 10.1016/j.jacadv.2024.100870

**Published:** 2024-02-21

**Authors:** Neil D. Patel

**Affiliations:** Division of Pediatric Cardiology, Children’s Hospital Los Angeles, Keck School of Medicine, University of Southern California, Los Angeles, California, USA

**Keywords:** Fontan, single ventricle, ventricular morphology

**Figure:**
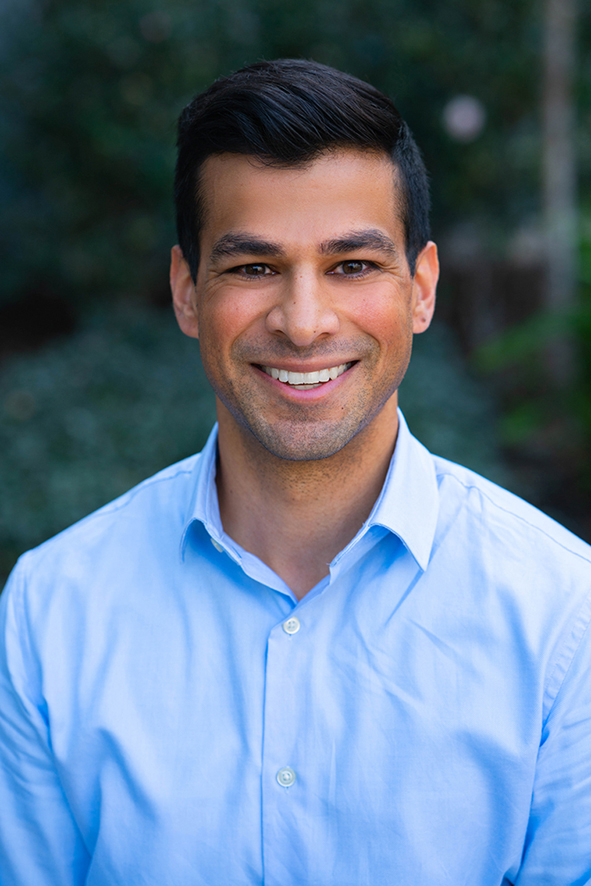

Patients with Fontan physiology are at risk for several long-term complications. Cardiac failure, chronic low flow, and venous hypertension place patients at risk for end-organ damage and significant morbidity and mortality.^1^, ^2^, ^3^ Ventricular morphology is often implicated as a predictor of outcomes. Patients with a single left ventricle (uLV) may have an advantage over those with a single right ventricle (uRV) with regard to exercise capacity, atrioventricular valve regurgitation, ventricular function, perioperative outcomes, and survival.^4^, ^5^, ^6^, ^7^, ^8^, ^9^, ^10^ Studies have sought to answer the uRV vs uLV question but are often limited to single-center cohorts with a short follow-up period and focus on transplant-free survival.

In this issue of *JACC: Advances*, Dib et al^11^ present a multicenter, retrospective cohort study comparing long-term outcomes of uRV vs uLV patients. The authors assessed a composite outcome consisting of sustained atrial arrhythmia, thromboembolic event, cardiac transplantation, and all-cause death following the Fontan operation. In total, the study included 384 patients across 12 centers. During a mean follow-up of 10.6 years, 89 events occurred. Patients with uRV had a >2-fold incidence of the composite outcome (3.7 vs 1.7 cases per 100 person-years) and remained independently associated with the outcome on the multivariate analysis.

Individual assessment of each of the outcomes demonstrated that uRV patients had a higher incidence of transplantation or death and atrial arrhythmias. The incidence of thromboembolic events was higher in uRV patients, though this did not reach statistical significance. The authors suggest that this higher incidence of thromboembolic events may be due to the increased risk of atrial arrhythmias in these patients. However, it is important to note that over one-third of the events were in the Fontan pathway or pulmonary arterial circulation. It is not known whether the systemic events were a result of atrial tachycardia, or other factors such as the presence of right to left shunts, anticoagulation practices, or Fontan flow dynamics. The total thromboembolism event rate of 8.3% emphasizes the need for improvement in anticoagulation practices for these patients.

One of the primary strengths of this study is its ability to demonstrate the adverse outcomes associated with uRV patients in a heterogenous patient population, across multiple centers, and with long-term follow-up. However, the increased risk of adverse cardiovascular events in uRV patients is not a surprising finding. Magnetic resonance imaging studies of Fontan patients have demonstrated that single RVs have a larger ventricular end-diastolic volume, lower mass-to-volume ratio, and higher end-systolic fiber stress, which underscore the notion that the RV is not intended to support the systemic circulation and adapts poorly.^12^ In additional to ventricular dysfunction, atrioventricular valve failure plays an important role in Fontan outcomes, and multiple studies have demonstrated a strong association between uRV patients and atrioventricular valve regurgitation.^5^^,^^12^^,^^13^

In conclusion, the study by Dib et al^11^ serves as an important reminder that not all Fontan patients are the same. It is well demonstrated by the authors that uRV patients are at significantly increased risk of adverse outcomes with comparison to uLV patients. In understanding these differences, many questions remain in the management of these complex patients.

## Funding support and author disclosures

The author has reported that they have no relationships relevant to the contents of this paper to disclose.
